# Food System Transformation and Gut Microbiota Transition: Evidence on Advancing Obesity, Cardiovascular Diseases, and Cancers—A Narrative Review

**DOI:** 10.3390/foods12122286

**Published:** 2023-06-06

**Authors:** Jasper Okoro Godwin Elechi, Rosa Sirianni, Francesca Luisa Conforti, Erika Cione, Michele Pellegrino

**Affiliations:** Department of Pharmacy and Health and Nutritional Sciences, University of Calabria, 87036 Arcavacata di Rende, Italy; rosa.sirianni@unical.it (R.S.); francescaluisa.conforti@unical.it (F.L.C.); erika.cione@unical.it (E.C.); michele.pellegrino@unical.it (M.P.)

**Keywords:** gut microbiota, food system, diet transition, non-communicable diseases

## Abstract

Food, a vital component of our daily life, is fundamental to our health and well-being, and the knowledge and practices relating to food have been passed down from countless generations of ancestors. Systems may be used to describe this extremely extensive and varied body of agricultural and gastronomic knowledge that has been gathered via evolutionary processes. The gut microbiota also underwent changes as the food system did, and these alterations had a variety of effects on human health. In recent decades, the gut microbiome has gained attention due to its health benefits as well as its pathological effects on human health. Many studies have shown that a person’s gut microbiota partially determines the nutritional value of food and that diet, in turn, shapes both the microbiota and the microbiome. The current narrative review aims to explain how changes in the food system over time affect the makeup and evolution of the gut microbiota, advancing obesity, cardiovascular disease (CVD), and cancer. After a brief discussion of the food system’s variety and the gut microbiota’s functions, we concentrate on the relationship between the evolution of food system transformation and gut microbiota system transition linked to the increase of non-communicable diseases (NCDs). Finally, we also describe sustainable food system transformation strategies to ensure healthy microbiota composition recovery and maintain the host gut barrier and immune functions to reverse advancing NCDs.

## 1. Introduction

The evolution of humans has witnessed constant transformation as the quest for civilization, globalization, and sustainability continues. It also has always stayed in the food system and the environment, as attempts are constantly made for food to meet the needs of the rapidly changing world and the growing population, projected to reach 10 billion by 2050. A very rich and diverse set of agricultural and food knowledge has been acquired through evolutionary processes, which can be characterized as systems [1]. A long-term historical perspective that has altered and become more sophisticated in accordance with economic progress can be utilized to investigate the concept of the food system. It consists of the following: (1) the chain of activities from producer to consumer; (2) the drivers and outcomes of the food chain, which have economic, political, environmental, health, and social dimensions; (3) the numerous entities, institutions, and people directly and indirectly involved; and (4) the connections between all of these elements, which means that an action in one area of the system has an impact on other areas of the system [2]. Braun et al. [3] conceptualized food systems as “the entire range of actors and their interconnected value-adding activities involved in the production, aggregation, processing, distribution, consumption, and disposal (loss or waste) of food products that originate from agriculture (including livestock), forestry, fisheries and food industries, as well as the broader economic, societal, and natural environments in which they are embedded”. A sustainable food system ensures that everyone has access to food security and nutrition while maintaining the economic, social, and environmental underpinnings necessary to provide food security and nutrition for future generations [3]. Thus, a sustainable food system must be economically feasible, provide substantial social benefits, and have either positive or neutral environmental effects [4]. Since the end of World War II (WWII), there has been a major rise in global food and agricultural production, which has been fuelled by a mix of population and economic development, as well as technological and cultural changes in production methods [1]. Food systems began to evolve when agriculture, including the domestication of animals, opened the door for long-term settlements. As a result, human society was altered; unlike previous hunter-gatherers, agriculturalists no longer needed to be constantly on the move in search of new food sources [5]. Grain cultivation made it possible to dry and store some of the harvest for later use. In each of the centres of civilization, distinct grain cultures developed: maize in Mexico, rice in China, and wheat and barley in the Middle East. The Green Revolution significantly influenced the dominant ideologies in contemporary agricultural practice and helped create intensive agricultural production techniques that have led to an overall rise in food demand and a shift in dietary preferences towards more resource-intensive foods. The overall food system, or the set of supply chains from farms through midstream segments of processing, wholesale, and logistics to downstream segments of retail and food service (restaurants and fast-food chains), has changed in parallel with diet changes [6]. The transformation is the tale of a “double-edged sword,” demonstrating its links to both positive and negative diet side trends, such as the rise in fast food and highly processed food consumption, as well as to parallel trends, such as the decrease in food costs, de-seasonalization, increased convenience of food preparation, which reduces the time required to do so, and increased availability of some nutrient-dense foods such as meat and dairy [6]. As a result, food systems have been evolving continuously since the dawn of agriculture, with each advancement bringing new advantages and challenges, as well as an increase in diversity and complexity [5]. A number of global challenges, ranging from diseases and poverty to environmental degradation, are concentrated on the current food system framework. Moreover, poor diet-related NCDs, such as cardiovascular disease (CVD), diabetes, and some cancers, are increasing globally, causing an estimated 41 million deaths annually, or 74% of all deaths worldwide [4,7]. It is not viable to simply extrapolate present production and consumption patterns to achieve the increase in food production necessary to meet expected future demand [1]. Recent historical tendencies of expansion and intensification will weaken the underlying resource basis upon which the food system is built. We are aware that diversified and resilient ecosystems, which are the cornerstone of human health and well-being, depend on a healthy world in a new global view identified as one health approach. A structural shift in the food system towards a more sustainable and resilient state is critical for ecosystem preservation as well as human population health in the future [1,4].

**Figure 1:**
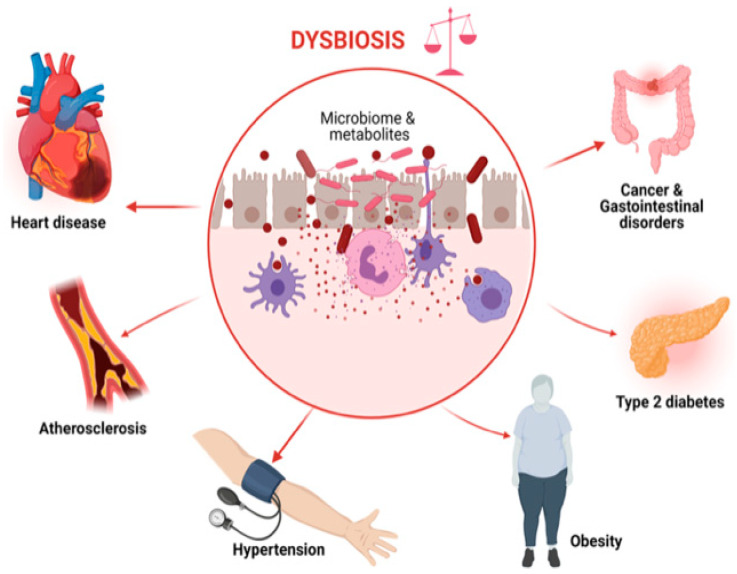
Host–microbiota interactions and their relationship with disease. Reprinted from Masenga et al. [27] under the terms and conditions of the Creative Commons Attribution (CC BY) license (https://creativecommons.org/licenses/by/4.0/).

The interactions of the changing food systems, diet transition, and environment have resulted in the alteration of microbiomes, leading to epigenetic changes and a global burden of health challenges. Several hundred distinct microbial species may be found inside humans, and they collectively have 150 times more genes than the human genome does [8,9,10]. The term “microbiota” refers to the collection of bacteria, viruses, fungi, and archaea that live in various bodily cavities and have co-evolved with people over the past six million years to develop a highly controlled symbiotic relationship. Each person has a unique microbiome composition that quickly changes throughout early development and becomes entrenched in maturity [11]. Genetic and environmental variables, such as nutrition, geographical location, exposure to toxins/carcinogens, and hormones, all affect changes in microbial composition [12]. More than 100 trillion microbial cells (or 10^13^–10^14^) make up the intestinal microbial population that lives in the human gut, with a microbial cell-to-human cell ratio of about 1:1. [13]. These figures are based on the total number of bacteria present in the colon (3.8 × 10^13^ microorganisms), the organ with the highest concentration of microbes [13]. *Firmicutes*, *Bacteroidetes*, and *Actinobacteria*, the three main bacteria taxa, make up most of the varied gut microbiota [14,15]. The development and differentiation of the mucosal immune system, the control of epithelial cell homeostasis and barrier integrity, and the coordination of systemic metabolic and endocrine activities are all influenced by gut microbiota [16,17]. Alterations in tissue and organ function are caused by the breakdown of mutualistic microbiome-host interactions in the gut, which may result in the beginning or development of illnesses. Many studies have shown that a person’s gut microbiota partially influences the nutritional content of food and that diet in turn shapes both the microbiota and the microbiome [18,19]. Long-term alterations in the gut microbiota transition that result in dysbiosis are connected to changes in the food system and some elements of the diet transition. Dysbiosis, a change in the composition of the intestinal microbiota, has been linked to a variety of chronic diseases, such as metabolic disorders (such as obesity and obesity-associated metabolic diseases such as type 2 diabetes mellitus (T2DM) and non-alcoholic fatty liver disease (NAFLD) [10,20,21], immune-mediated diseases such as inflammatory bowel diseases (IBD) [22,23], and colorectal cancer (CRC) [24,25] (Figure 1). According to estimates, the world economy will suffer losses from obesity, malnutrition, and NCDs totalling USD 760 billion, USD 3.5 trillion, and USD 7 trillion, respectively [26].

The composition of the gut microbiota, which directly affects host homeostasis and biological processes, is significantly influenced by diet. With innovative dietary practices, the vital mutualism between the human host and its bacterial symbionts may change, possibly affecting the immune system and intestinal barrier functions [28]. For example, throughout evolution, the primary source of food for primates was plants [29]. This suggests that certain species and metabolic functions within gut bacterial ecosystems, as well as individuals able to benefit from their presence and functions, may have been preferentially selected [30,31,32,33]. Therefore, the evolution of primate-microbiota symbiosis may have shaped the genetic structure of the host immune system and digestive tract to enable a tolerogenic response towards microbial ecosystems suitable for ecological niches created by a significant amount of plant consumption, as well as to respond appropriately to stimuli generated from those, in order to ensure their containment inside the gut [34,35,36,37]. Therefore, it can be believed that a food system dietary setting composed primarily of ripe and unripe fruits, young leaves, flowers, seeds, and, at times, roots and tubers would have chosen the most suitable individuals to tolerate microbial species able to create ecosystems equipped with enzymatic repertoires adapted to the degradation of the majority of the non-digestible elements of this diet [29]. In fact, a sizeable amount of the health advantages of a plant diet are linked to the modification of the gut microbiota [38,39]. Preservation of the gut barrier function is crucial for preventing uncontrolled permeability and bacterial translocation [35,40,41]. This is accomplished by the production of short-chain fatty acids (SCFAs) by gut microbes from the fermentation of non-digestible carbohydrates [40].

**Figure 2:**
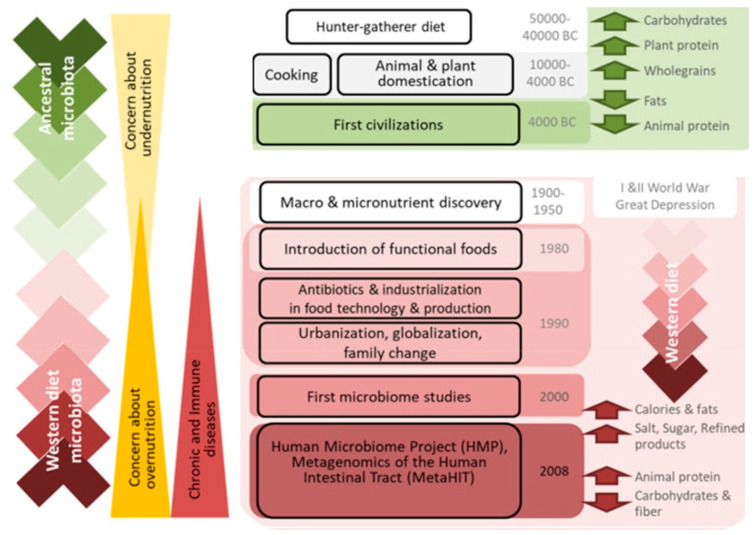
Drivers of dietary trends and their relation to microbiota composition and changes in human health. Adapted from Moles and Otaegui [55]. under the terms and conditions of the Creative Commons Attribution (CC BY) license (https://creativecommons.org/licenses/by/4.0/).

However, evidence from humans and nonhuman primates suggests that the so-called “Western diet (WD),” which is characterized by a high caloric intake, a high concentration of animal proteins, fats, and monosaccharides, and a low intake of fibre, promotes the pathogenesis of immunometabolic abnormalities as well as dysbiosis [42,43]. Therefore, assuming that the primate host is designed to tolerate specific microbial ecosystems, it can be assumed that adopting a WD, which should result in a shift towards microbial ecosystems for which the host immune system is not designed to provide a tolerogenic response, could trigger a local inflammatory response, which in turn would intensify dysbiosis [44,45,46] (Figure 2). Additionally, these novel microbial ecosystems’ inappropriate stimuli would impair the gut’s ability to act as a barrier against pathogens, increasing the number of pathogens and pathogen-associated molecular patterns that enter the bloodstream, a condition known as metabolic endotoxemia [47,48,49,50]. If gut homeostasis is not restored, this may trigger the beginning of low-grade systemic inflammation, which can become chronic [51,52]. According to this caussal chain of pathophysiology, immunometabolic dysregulation is mostly caused by the primate host’s poor adaptation to Western-induced microbial habitats. BaAka and Bantu microbiotas were found to be more comparable to one another than to those of Westerners when compared to coexisting Bantu agriculturalists, US Americans, and BaAka hunter-gatherers [53]. However, similarities between the Bantu and American microbiota profiles were found, indicating that agriculture may have gradually sparked a loss of indigenous microbes that has since accelerated in industrial societies over the past few centuries, concurrent with this westernisation of the human nutritional environment [54].

The goal of the current narrative review was to explain how changes in the food system over time affect the makeup and evolution of the gut microbiota, which in turn advances obesity, CVD, and cancer. First, we briefly outline the food system’s variety and gut bacteria’s functions. Second, after defining the functions of the gut microbiota and the variety of the food system, we concentrate on the link between the development of the food system transformation and the transition of the gut microbiota system to shape the evidence of rising NCDs and, finally, sustainable food system transformation strategies to ensure the recovery of a healthy microbiota composition and to maintain host gut barrier and immune functions to reverse the advancing NCDs were described.

## 2. Materials and Methods

The relevant studies to be included were searched in the PubMed, Scopus, Google Scholar, and Web of Science databases from January to March 2023. The search terms used alone or in combination were: “Food systems” “OR “food system transformation” OR “gut microbiota” OR “microbiota transition” OR “obesity” OR “obesity and food system” OR “obesity and microbiota transition OR “cancer” OR “cancer and food system” OR “cancer and microbiota transition” OR “cardiovascular diseases” OR “cardiovascular diseases and food system” OR “cardiovascular diseases and microbiota transition” OR “dietary pattern” OR “dietary pattern and microbiota”. The selection of articles for inclusion in the review was undertaken in two stages. The first stage involved screening the titles and abstracts of the search results against the eligibility criteria. In the second stage, the full articles of papers selected in the title/abstract screening stage were screened to confirm that they met the eligibility criteria.

## 3. Evolution of Food System Transformation and Gut Microbiota System Transition

**Figure 3:**
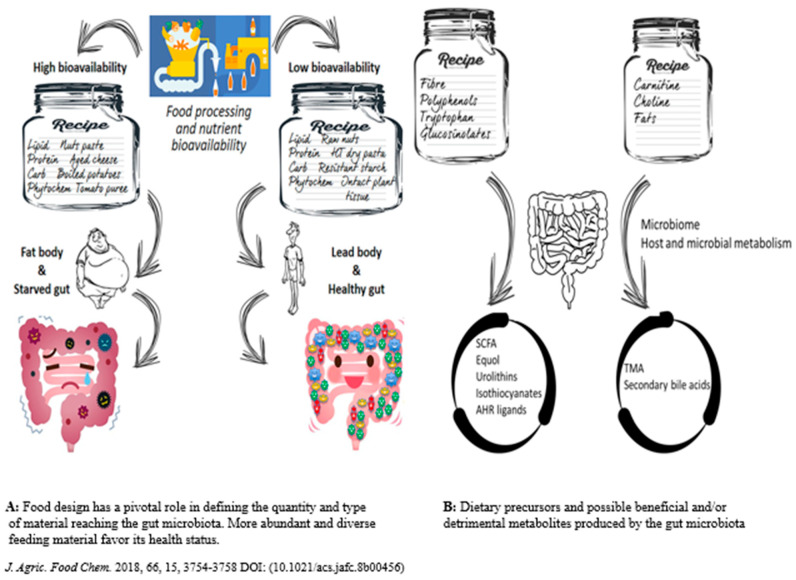
Interactions of the food processing system and gut microbiota transition. Reprinted from Ercolini and Fogliano [66]. under the terms and conditions of the Creative Commons Attribution (CC BY) license (https://creativecommons.org/licenses/by/4.0/).

Natural selection developed the dietary needs of humans over millions of years, while humans and their hominid predecessors only ate meals from a menu of wild animals and uncultivated plants [56,57]. Vertebrates have been able to occupy various environments and use various eating techniques because of variations in the length and compartmentalization of their digestive tracts over time. The requirement to maximise two fundamental biological processes—nutrient absorption and microbial fermentation of slowly digesting plant foods—led to many of these advancements in gut physiology [57]. Understanding nutritional history has been the focus of anthropological study for decades, partly because dietary changes were probably linked to significant anatomical and cultural changes (e.g., the growth in relative brain size and the birth of modern society via agriculture). The diversity of the human gut microbiota is thought to be largely influenced by dietary practices [1,58]. As a result, host-bacterial connections are a long-term result of diverse co-evolutionary processes, where favourable interactions between the microbe and the host are a result of their shared advantages [59]. While food processing has been a necessity for human society since the dawn of time to ensure the security, palatability, digestibility, and safety of food products, recent socio-economic developments have given rise to food items and dietary habits that raise concerns about potential health effects [60,61] (Figure 3) (Table 1). A variety of physical, thermal, and chemical methods, including milk centrifugation, sterilization, and oil bleaching, have been developed since the 19th century, beginning with canning and pasteurization. The management boards of the food sector, particularly after the World Wars, turned their attention to customer pleasure while pursuing profit and convenience [62]. In addition, since the 1980s, our food system has become ever more international, with huge multinational corporations distributing crops and goods worldwide [63,64]. These innovations have given rise to a variety of highly processed foods that are typically very tasty and affordable but have high calorie counts, high sugar, fat, and salt levels, as well as minimal fibre and are extremely sterile, which means that they have the fewest microorganisms possible for preservation [62] (Figure 3). The basic idea is that microorganisms in the stomach thrive on what the human body cannot use [65]. In other words, the actual food for commensal bacteria in the stomach is inaccessible dietary components. Figure 3A exemplifies this notion. A food processing design that decreases bioaccessibility may be helpful to the host in the setting of an excess of macronutrient consumption and high-calorie-dense meals [66]. It is well recognized that a plentiful supply of various meals enhances biodiversity in the microbiota, as well as the range of microbial genes produced that might be health status triggers. Different dietary precursors can be transformed into helpful or harmful metabolites by gut microbiota members [66], as shown in Figure 3B.

A well-balanced and healthy gut microbiota composition results from a rich and diversified microbial population [67]. More than 500 species from 6 phyla—*Actinobacteria*, *Firmicutes*, *Bacteroidetes*, *Proteobacteria*, *Fusobacteria*, and *Verrucomicrobia*—make up the human gut microbiota, and 90% of this community of bacteria are *Bacteroidetes* and *Firmicutes*. More than 200 distinct genera, including *Lactobacillus*, *Bacillus*, *Clostridium*, *Enterococcus*, and *Ruminococcus*, make up the *Firmicutes* phylum. In total, 95% of the *Firmicutes* phylum are made up of the *Clostridium* genera. The predominant genera in the genus *Bacteroidetes* include *Bacteroides* and *Prevotella* [28]. Nevertheless, compared to *Firmicutes* and *Bacteroidetes*, *Proteobacteria*, *Actinobacteria*, and other pylas of *Verrucomicrobia*, *Cyanobacteria*, *Fusobacteria*, and *Spirochaetes* are rare in the colon [28,68]. The *Fusobacterium* is linked to several human disorders. As a result, it is frequently referred to as a pathogenic bacterium. *Firmicutes* and *proteobacteria* are also regarded as harmful since they harm the gut’s metabolism of glucose and fat. *Verrucomicrobia*, *Actinobacteria*, and *Bacteroidetes*, in contrast, have a positive impact on gut health by helping the host develop resistance to infectious disease, contributing to glucose homeostasis, and producing SCFAs that are known to reduce inflammation [68]. The distribution of gut microbes, whether symbiotic or dysbiotic, strongly correlates with a higher risk of disease or optimum health. According to studies, the symbiotic microbiota is dominated by symbionts like *Bacteroidetes* the *thaiotamicron*, *Bifidobacteria*, *Lactobacilli*, and *Faecalibacterium prausnitzii*, while the dysbiotic microbiota is dominated by pathobionts such as Bacteroides spp. and Clostridium difficile [69].

Here, we examine the historical evolution and transition in the food system and gut microbiota to investigate variance in gut microbiota and comprehend how these bacteria may have co-evolved with humans. According to the technological perspective, the food system may be classified as pre-agricultural, agricultural, agri-industrial, and most recently agri-tertiary era, in that sequence [70]. The framework put forth by Gaitán-Cremaschi et al. [71] categorizes and maps the diversity of current food systems in terms of their transitional pathways, i.e., dominant food systems supported and aligned to the food system regime, niche food systems, and hybrid forms, based on a set of structural characteristics. They have various configurations depending on how these structural requirements are connected to the food system regime, niche food systems or hybrid forms. The distinction between three interrelated food system components—(i) the agricultural production system, (ii) the value chain, and (iii) the structures that support innovation and the regular operation of agricultural production systems and value chains—is a recurring theme in various attempts to characterize food systems [71]. The five historical food systems identified by Renske [72] through the article of Paul Matteucci (foodcrunch.com) include Food System 1 (the hunter-gatherer approach to food), Food System 2 (the transition from nomadic life to settlement and the development of agriculture), Food System 3 (selection of desirable traits in plants and animals and optimizing of food production for taste, climate, and pest protection), and Food System 4 (agricultural production for taste, climate, and pest protection). Lastly, there is a proposal for the sixth food system—one that is optimized for the integrated and comprehensive priority of planetary and human health [72]. Here, we briefly discuss each system and the dynamics of the gut microbiota in food systems transition.

### 3.1. Food System 1 (Hunter-Gatherer)

Around 7–8 million years ago, humans and other great apes last had a common ancestor, according to Kunimatsu et al. [73]. Most of the calories consumed by chimpanzees (*Pan troglodytes*)—our closest living relatives—come from fruit [74,75]. Moreover, a minor portion of their diet consists of animal products, such as insects and monkeys. Early hominid diets diverged from apes’ forestry diets to include a wider variety of foods found in open areas [75]. As a result, the earliest food system for humans, known as Food System 1, has existed for at least hundreds of thousands of years. The term “hunter-gatherer attitude to food” is frequently used to describe this [72]. As humans have developed, they have eaten a variety of wild animals and plants that thrive in different bioregions worldwide [76]. Up until roughly 12,000 years ago, this system seems to have served humans quite effectively [72]. By softening food texture, increasing calorie density, and lowering toxins at this time, the employment of cooking methods and the discovery of fire aided in the evolution of human GI physiology [77]. The “expensive tissue theory” [78,79,80] states that a decrease in the size of an energetically costly GI tract results in an increase in the size of an energetically expensive brain, which in humans may have been facilitated by improvements in diet [80].

Hominids’ food preferences and nutritional flexibility significantly increased between 4.4 and 2.3 million years ago [81]. Early hominid development saw our ancestors migrate from the forest to different environments, including the savannah, dramatically diversifying their food. The introduction of agriculture is thought to have eventually led to their diet containing more animals, fermented foods, and, most recently, significant amounts of starch [75]. The bulk of the daily protein consumed by great wild apes comes from forest plants, particularly young leaves [82]. Nonetheless, both early and contemporary humans preferred eating proteins in a form that was a little easier to digest, like meat [75,83].

### 3.2. Food System 2 (Neolithic Revolution)

The so-called “Neolithic revolution”, which started some 10,000 years ago with the development of agriculture and animal husbandry, brought about significant changes in nutrition and lifestyle circumstances [84]. It is unclear what need people were addressing at the time of the shift, but it is apparent that they were under pressure to abandon their nomadic way of life in favour of settling down and developing an early form of agriculture, which is what Food System 2 is [72]. The development and expansion of agriculture and animal husbandry mark the beginning of a significant change in human diets [57]. The transition from hunting and gathering to agriculture, which involved the domestication of animals and plants offering more calories than nondomesticated plants, was a crucial step in the evolution of humans, which caused the dietary pattern to concentrate more on a small selection of foods and reduce the diversity of nutrients [80]. Further dietary shifts have occurred as a result of the development and expansion of global food production. People now concentrate more intently on the intake of a few basic foods because of mass food manufacturing. The host GI tract shape and long-term dietary history limit the establishment of a preserved and stable microbial community [1,85]. Following this, food resources increased and were more reliable. Population densities that are orders of magnitude higher than those feasible under hunter-gatherer subsistence economies are the result of the food production and storage technology connected with this dietary transition [57].

### 3.3. Food System 3 (The Advent of Agriculture)

Later, depending on the seeming need for “better” food, people discovered that they could breed plants and animals with the features they wanted. This allowed us to control pests, adapt to different climates, and maximize food production for flavour. Because human taste receptors are among the finest monitors of nutrients, flavour selection is highly crucial [75]. The work of Gregor Mendel in the middle of the nineteenth century is the greatest example of this system, which was known as Food System 3 [86]. In addition, with the development of agriculture, basic foods with which the hominid genome had limited evolutionary experience were introduced. As a result, our diet underwent a substantial transformation eight to ten thousand years ago, with grains and carbohydrates playing a much larger role. This led to continued shaping and evaluation of the human gut microbiota to be suitable for the new food environment, thereby affecting the optimal health of the host with the onset and proliferation of dietary-related NCDs.

### 3.4. Food System 4 (Industrial Revolution and the Green Revolution)

At the end of World War II, vast ammonia manufacturing facilities that had previously been used to make explosives needed to be put to new uses, especially in the United States. We also discovered that petroleum-based agriculture had significant labour-saving potential, both in the production of herbicides and fertilisers, as well as in the use of agricultural machinery. Around this time, we no longer chose plants based on flavour, but rather on yield, including insect resistance [72]. This gave birth to a new food system—Food System 4. More importantly, food processing techniques were created, especially after the industrial and green revolutions of the post-World War II era, which enabled the development of energy-dense, nutrient-poor, largely sterile foods as major dietary components in developed economies. These foods were not previously encountered during the evolution of hominids [57], as they mark a significant departure from the essential component of dietary fibre.

The quantity and quality of dietary fibre is a key food macronutrient that distinguishes ancient and hunter-gatherer diets from our modern diet [57]. While the current recommended daily intakes in the United States are 20 to 30 g/day and the typical American consumption is between 10 and 20 g/day, it has been estimated that the Paleolithic diet provided more than 100 g/day of fibre [87]. It is also conceivable that the fibres making up the greater majority of the present food intake will differ in quality or chemical composition. In contrast to the much more varied collection of fibres from a wide range of fruit, vegetables, roots, legumes, and nuts, which hunter-gatherers would have consumed and in Paleolithic times, a high percentage of dietary fibre in the modern diet is derived from cereal grains [57,84,88]. Compared to fibre obtained from cereal bran, these foods have greater concentrations of fermentable carbohydrates, such as resistant starch. A significant portion of the fermentable carbohydrates in the diet come from resistant starch. The colon’s energy economy is driven by fermentation, which also influences the relative abundance of the local saccharolytic bacteria and the synthesis of SCFAs, such as acetate, propionate, and butyrate. The host may be able to absorb and utilise up to 95% of the SCFAs generated in the colon. Similarly, SCFAs generated in the colon may function in regulating gastrointestinal peptides and hormones that affect satiety. They also play a key role in strengthening the intestinal barrier and lowering mucosal permeability, which affects systemic inflammation [57].

### 3.5. Food System 5

We have finally transitioned to a food system that is optimised for shelf life, convenience, and logistics, but only in the last thirty to forty years, based on the necessity and desire for enhanced efficiency. For market actors with the ability to influence the system in their favour, such as vertically integrated producers of animal protein, manufacturers of commodities, and distributors of processed goods, this system has also shown competence at maximizing economic returns. However, in terms of their capacity to derive profit from the system, this structure has traditionally neglected the real farmers, ranchers, and food producers [72]. Consumers’ capacity to make decisions that are in line with values-based procurement has been hampered by the growing consolidation and commodification within Food System 5 [72]. This paradigm has created a number of new problems, including processed foods that are deficient in vitamins, minerals, and other crucial components for human health. It is believed that food flavour is a new ingredient added during processing. The nutrients that make the flavour that our taste receptors have evolved to perceive have largely been disregarded, since flavour production is regulated independently from food production. Because of this, many foods today are deficient in the nutrients people need to flourish [72]. Convenience and cost-effectiveness in the supply chain function well together, but the nutritional density and overall healthfulness of foods suffer significantly [89]. To that purpose, the modern food manufacturing method increases productivity, packs in calories, extends shelf life, and provides food to the general public with the highest convenience. Nonetheless, general suppliers, policymakers, and consumer consciousness are largely oblivious to the implications of such convenience [72].

Studies on intestinal microbiota raise concerns about whether eating a hyper-hygienic, highly processed diet today reduces the functional maturity of the microbes in the body by preventing the transfer of advantageous genes between the gut microbiota and microbes from the diet and environment [80]. Moreover, gut microbiota may be significantly impacted by the rising use of sanitization and antibiotics in food processing in Food System 5. Since food firms compete for market share and cost savings culture, the global food industry, and the media all have an impact on an individual’s attitudes, taste preferences, and dietary habits, which in turn have an impact on the activity and composition of the gut microbiome [80]. Additionally, there is mounting evidence that, since industrialization, the human diet has undergone a sudden, profound simplification that occurred too recently on an evolutionary time scale for the human genome to adapt to the efforts to feed 10 billion people by 2050 [90,91]. The underlying evolutionary genesis of “civilization disorders”, including CVD, obesity, and cancer in the twenty-first century, has been proposed to be this maladaptation to the contemporary diet [90,91].

### 3.6. Food Systems 6 (The Birth of a New Food System)

Renske [72] also suggested that a new food system, known as food system 6, should emerge. The sixth food system for mankind, one that is optimised for the integrated and comprehensive priority of planetary and human health, has to be created, the author claims. It is time to redesign our existing food system. A holistic perspective on the vitality of farms, sustainable ecosystems, healthy communities, and justice and fairness will be included in this system, which must take into account the interactions between all food system stakeholders. These are the aspects and components of food production that Food System 5 ignores. The food sector should avoid these fleeting trends and instead concentrate on finding answers to the issues perpetuated by our current food system. The emphasis currently largely remains on immediate consumer satisfaction rather than mindful process and sustainable product development, as grocery store shelves are crowded with trendy products such as “gut-healthy foods”, protein substitutes, or a “healthier” version of an established brand [72].

### 3.7. The Dynamics of the Gut Microbiota System in Food System Transition

As seen above, dietary changes in humans over time are likely to have happened in stages, starting with an increase in the sharing of plant roots, bulbs, and tubers in early Homo species [92,93] and continuing with an increase in meat consumption in Homo sapiens during the Pleistocene before being adopted by agriculture and the domestication of animals almost 10,000 years ago (Table 1). According to histories and lifestyles, dietary changes cause distinct changes in gut microbiota that may be used to classify human groups [94]. As a result, nutritional change under different food systems throughout human history has been crucial to human evolution, and as a result, the gut microbial population has co-evolved with the host over time [95,96,97]. Horizontal gene transfer plays a role in the coevolution of the gut microbiota with its host [98] to acquire function and adapt to new environmental circumstances. Comparative genomic research conducted more recently [99] showed that horizontal gene transfer often occurs in the human gut microbiota. As a result, horizontal gene transfer adds to the complexity of the gut microbiome’s metabolic process and enables both the host and its resident bacteria to adapt to changing environmental conditions. Thus, a host’s capacity to adapt to environmental changes is determined by both the co-metabolic skills of the host and the gut bacteria [80].

The Western diet (WD), which is based mainly on Food Systems 4 and 5, exerts significant evolutionary pressure on the gut microbiota due to the decrease in intake of complex carbs and fibre across all food systems and the increase in consumption of simple sugars. Lopez-Legarrea et al. [100] stated that *Bifidobacterium* and certain Clostridium subgroups (*Roseburia* and *Eubacterium rectale*) showed a substantial decrease in reduced or restricted fibre intake that was directly associated with the reduction in butyrate levels in stools. Since lactic acid bacteria, especially Lactobacillus, and the species *Faecalibacterium* of Clostridium cluster IV are productive microorganisms [101,102], a rise in the population of these bacteria is caused by a high intake of ultra-processed foods. Similar to how the genus Firmicutes increases significantly in WD, the genus Bacteroidetes decreases. Bacteroidetes, unlike Firmicutes, have an enzymatic system for the host’s non-digestible polysaccharide metabolism, which promotes their growth and survival [102]. The conditioning of WD substrates causes the loss or reduction of Bacteroides, which causes dysbiosis and the loss of several specific microbial niches in the intestine. As a result, differences in microbial phylogenies have been observed in people with chronic metabolic diseases [103]. The large proportion of saturated fats in WD is another distinguishing factor. High-fat meals also have an impact on how the gut’s bacterial population is regulated, resulting in a 50% decrease in the population of the bacteria *Bacteroides*, *Verrucomicrobia*, *E. rectal*, *C. coccoides*, and a proportional increase in *Firmicutes* and *Proteobacteria* [102]. Moreover, it promotes hyperinsulinemia and excessive lipid accumulation in the liver and adipose tissue by inducing the production of pro-inflammatory cytokines (IL-1, IL-6, and TNF-a). A lower number of *Bifidobacterium* and a greater quantity of plasma endotoxin (LPS formed from gram-negative bacteria) have been linked to a high-fat diet, leading to low-grade inflammation and the emergence of metabolic disorders [104,105].

Research that compares the gut microbiota of individuals from various geographic areas and with various food systems offers evidence for the importance of dietary habits in shaping the characteristics of gut microbial composition [106]. For instance, the Hadza of Tanzania have more microbial variety and richness in their gut microbiota than people from Italy [107]. Similarly, healthy children from Bangladesh also have more diverse bacterial populations in their distal guts than children from the United States [108]. Compared to European children, the gut microbiota of African children exhibits a higher abundance of Firmicutes and a lower abundance of Bacteroidetes [94]. Notably, specific SCFA-producing bacteria, such as *Xylanibacter*, *Prevotella*, *Butyrivibrio*, and *Treponema*, enriched in the guts of African children may be the result of their typical dietary habits (low in fat and animal protein and rich in starch, fibre, and plant polysaccharides), and these bacteria may aid local people in maximizing the energy intake from indigestible plant components [94]. Comparing rural Papua New Guineans, Malawians, and Amerindians to US residents also reveals notable changes in the faecal microbiota makeup [109,110]. Similarly, in Japanese communities that regularly consume uncooked, non-sterile seaweed, the genome of the human gut symbiont *Bacteroides plebeius* has retained -porphyranase, an advantageous enzyme that can digest algal cell walls from *Zobellia galactanivorans* [80,111]. In fact, a WD and a sedentary lifestyle have been linked to low microbial complexity (or gene richness), which may in turn lead to illnesses linked to excessive weight gain [112]. These findings suggest that there are significant differences in the gut microbial composition of individuals in developing and developed countries. Dietary effects as a result of the type of food system may significantly contribute to this variation [106], as well as the diversity of the gut microbiota [113] and even significant epigenetic changes [39,114].

## 4. The Nexus of Food System and Gut Microbiota Transition in NCDs

The hunter-gatherer lifestyle is defined by the reliance on a variety of wild (undomesticated) animal and plant sources, which also helps to explain the adaptive series of changes that marked the birth of our genus. It became common in public health to refer to our hunter-gatherer past to explain the rise of NCDs (diabetes, obesity, cancer, and heart disease) in the developed world as epidemiology developed throughout the 20th century alongside a growing understanding of our species’ evolution [115]. It is now widely established and accepted in public health that modern, industrialised surroundings are fundamentally different from the habitats in which humans originated and that these most recent changes cause disease. According to De Filippo and Lionetti [57], the epidemic of obesity ravaging people in both developed and developing nations, as well as the steep rise in illnesses of affluence (type 2 diabetes, coronary vascular disease, NAFLD, and certain cancers), may be the most obvious effects of recent divergence from nutritional symbiosis with fermentative gut microbiota. Our hunter-gatherer ancestors were not used to being obese, and given the chronic illnesses obesity is connected with, our bodies are not equipped to handle it [57]. These findings imply that dietary alteration of the gut microbiota affects the processing of microbial-host co-metabolic reactions, as well as the risk of developing metabolic diseases. It also suggests that fermentable carbohydrates might alter these diet-microbiota interactions. The notion connecting contemporary food and lifestyle to an increased risk of metabolic illness is supported by observations in current hunter-gatherer groups; in particular, in Australia, Aboriginal people who adopt the Western-style diet also experience an increasing prevalence of type 2 diabetes. Remarkably, this scenario is rectified when these cultures adopt their more traditional diets, which are rich in fermentable carbs, rather than the Western-style diet [57,116]. Similar to this, adjusted risk models show that the Pima Indians in Arizona who adhere to a traditional desert-type diet high in whole-plant foods and particularly elevated in fermentable carbohydrates have a 2.5 times lower risk of developing diabetes than the same ethnic group who adhere to a modern “Anglo”-type diet typical in America, which is low in fermentable fibres [57,117]. For example, traditional populations have a higher genetic propensity for obesity and diabetes than modern populations, such as those in the Americas and the Indian subcontinent, with the “thrifty gene” theory postulates that genetic selection for energy harvesting and storage from diets low in available energy contributes to metabolic disease risk in these populations once they adopt high-energy, Western-style diets [118,119,120].

### 4.1. Food System and Gut Microbiota Transition in Obesity

The definitions of overweight and obesity include abnormal or excessive fat build-up that might harm one’s health. In their study of the growth in obesity from the Paleolithic to the Industrial Revolution, Keneilwe and Ontefetse [121] showed how eating habits changed over time. The low frequency of obesity among hunter-gatherers now and in recent history suggests that excess body fat was uncommon throughout our evolutionary history, with its incidence and prevalence throughout the Paleolithic epoch unclear [115]. Neolithic humans saw changes in food and dietary patterns, as well as indications of changes in microbiota with the development of agriculture and animal husbandry [122]. Similarly, the Industrial Revolution of the 1850s made processed goods, such as white sugar and flour, easily accessible [123], with increasingly sedentary work [124]. According to Keneilwe and Ontefetse [121], these modifications would cause energy to be conserved, which would then be converted to fat and result in obesity. As a result, there has been a drop in physical activity and an increase in the consumption of foods that are high in energy but poor in vitamins, minerals, and other micronutrients. These foods are also heavy in fat, salt, and sugar. During the past 40 years, obesity prevalence has grown significantly worldwide, rising from less than 1% in 1975 to 6-8% in 2016, from 3% to 11% in males and from 6% to 15% in women during the same period [125]. The middle-of-the-road scenario by Bodirsky et al. [126] shows an increase in the number of overweight and obese people from 1993 million (29%) in 2010 to 4135 million (45%) in 2050 and 5018 million (56%) in 2100. According to a recent report by the World Obesity Federation, more than half the world’s population aged 5 and above—51%, or more than 4 billion people—are projected to be overweight or obese by 2035. By comparison, 2.6 billion people worldwide (38% of the population) are overweight or obese to 24%, or nearly 2 billion people, by 2035. According to Bodirsky et al. [126], the absence of behavioural change will result in a pandemic level of weight gain and obesity in the future. This future route has a significant negative impact on public health and is in direct contrast to the sustainable development goal 2 (SDG2) aim of eradicating all types of malnutrition.

Major global changes in agro-industrial systems can be blamed for the development of a so-called Western dietary pattern, which has been strongly linked to the obesity problem [62]. Since 1850, but especially since World War II, there has been an increase in the production and consumption of sucrose, high-fructose corn syrup (HFCS), and vegetable oils. Additionally, labour-intensive milling and sieving of grains have produced highly refined flour devoid of fibre or germ, altering its nutritional value, and the practice of feeding cattle grain instead of grass results in meat with higher saturated fat contents than would be possible from wild or pasture-fed animals [62,84]. Western-style food products have emerged as a result of these developments, which are typically (very) processed, high in calories, high in saturated fat, added sugar, and salt, and low in fibre [60,84]. Many features of an obese gut microbiome have already been identified, and research into the relationship between the present obesity epidemic and gut microbiota, as well as the various processes involved, is currently increasingly popular. It has been demonstrated that an obese person’s gut microbiota is disrupted or changed in a number of ways. For example, it has been discovered that obese phenotypes may carry more distinct microbial communities than lean morphologies and that the obesity phenotype [62,127] may be passed on through the gut microbiota [128]. In other words, it is well accepted that an obese person’s gut has a less diversified microbial environment than a person of normal weight [129,130,131]. Although this shift has not been discovered in all studies, several researchers have discovered that obese people, including both mice and humans, carry more Firmicutes and fewer Bacteroidetes species [85,130]. What is more significant, however, is how the gut microbiota of an obese person changes to extract more energy from their diet and influence other pathways (including incretin production, gut motility, low-grade inflammation, etc.), which may help explain how the gut microbiota contributes to the development of obesity [132]. For instance, research on germ-free and Ob/Ob mice has revealed that an obese phenotype is linked to increased energy harvest in the gut. According to the authors [130,131,133], this is due to the production of SCFA by the microbiota from indigestible dietary components, most frequently carbohydrates, and subsequent absorption by the gut.

Nonetheless, SCFA synthesis by gut bacteria is typically seen as advantageous for gut and general health because of its gut barrier-strengthening [134], anticarcinogenic (butyrate), antidiabetic, and anti-inflammatory qualities [129,130,135]. There are additional reports of their participation in the production of satiety signals by adipocytes (leptin, adiponectin) [129], which would help prevent the onset of obesity, and by the gut’s endocrine L-cells (GLP-1, PYY, GIP). Moreover, processed foods, which make up a large portion of the WD, are frequently deficient in indigestible carbs, which produce SCFA and provide additional energy [62].

**Figure 4:**
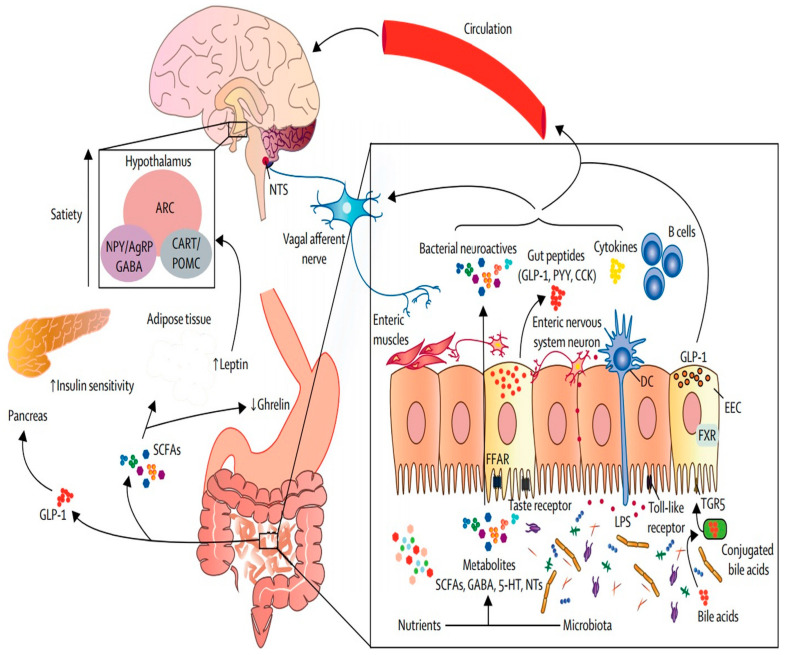
Link between obesity and gut–microbiota–brain axis. “Reprinted from Lancet Gastroenterol Hepatol, 2 (10), Torres-Fuentes et al. [146], The microbiota-gut-brain axis in obesity, 747–756, Copyright (2017), with permission from Elsevier”. Also Lancet special credit–“Reprinted from The Lancet, 2, Torres-Fuentes et al. [146], The microbiota-gut-brain axis in obesity, 747–756, Copyright (2017), with permission from Elsevier”. (License Number: 5555560985483).

The gut-brain axis influences several areas of physiology, including glucose balance, eating control, gut motility, and hunger. Therapeutics for a variety of disorders, including T2DM and obesity, have been investigated using this method [136]. A vast and complicated network of neurons and hormones communicates bilaterally between the gastrointestinal system and the brain, and their receptors control hunger, food intake, and obesity [137]. The presence of nutrients in the gastrointestinal system triggers complicated hormonal and neurological signalling to the brain, which is mediated by the vagus nerve [136]. Effector fibres transfer information from the gut to the NTS and to the smooth muscles of the gut [138] (Figure 4). The hypothalamus receives information from the NTS and uses it to control appetite, food intake, and energy balance in the neurons of the arcuate nucleus (ARC). Cocaine- and amphetamine-regulated mRNA, agouti-related protein, orexigenic neuropeptide Y, anorexigenic peptides (LEP), and pro-opiomelanocortin neurons make up the ARC [138]. Studies have demonstrated that vagotomy reduces anorexigenic hormone signalling, which results in increased food intake and weight gain in animal models [136,139].

Because certain gut bacteria can influence the release of gut hormones, such as GLP-1, ghrelin, PYY, and LEP, hypothalamic neuroendocrine pathways influence hunger and fullness [140]. Microbiota-derived SCFAs can bind to receptors on EECs and change the release of enteric hormones into systemic circulation [141]. Furthermore, activation of several taste (bitter, fat, umami, and sweet) receptors in EECs stimulates ghrelin, GLP-1, and cholecystokinin production [142]. The major SCFA released by gut bacteria, acetate, lowers appetite through central hypothalamic pathways [143]. The efficiency of calorie intake from consumed meals is increased by obesity-associated bacteria [136,144]. Therefore, compared to a lean-associated gut microbiota, an obesity-associated microbiota gives the host more energy from more indigestible carbs and proteins by increasing the synthesis of several primary fermentation enzymes and nutrient transporters [145] by increasing the synthesis of several main fermentation enzymes and nutrient transporters and increasing the amount of energy that the host receives from other indigestible carbohydrates and proteins [145,146].

### 4.2. Food System and Gut Microbiota Transition in Cancer

As there is ample evidence of cancer throughout the archaeological record, it is not a sickness of the contemporary day [147]. Nonetheless, it appears that cancer rates have progressively risen over the past century [148,149]. Although there is little information on the prevalence of cancer among hunter-gatherer communities, what information there is indicates that their rates of cancer and other chronic illnesses were much lower than those of contemporary people [150,151]. Worldwide, cancer is a leading cause of death [152,153]. The number of cancer patients in the United States of America (USA) alone has dramatically grown over the past 10 years, from 13.8 to 18.1 million, placing a USD 158 billion economic burden on the country [154,155]. According to projections made by Agrawal [156] and Akbar et al. [155], there may be 24 million active cases annually by the year 2030. Thus, it is wise to study our ancestors’ histories to learn how to avoid leading lifestyles that may encourage the development of chronic illnesses like cancer. As some mismatches are known to cause cancer, society has already started to move away from them [155]. The extent to which our contemporary diets contribute to the onset of cancer is still unknown. Every macronutrient consumption has been linked to the onset of cancer, depending on the type [155].

Bacteria can use a number of dietary and digestive components in the GI tract to create anti-cancer metabolites and possible oncometabolites [157]. A genotype more suited to digesting complex carbohydrates from plant-based diets has been established after 10,000 years of agrarian (farmers and pastoralists) existence and the Industrial Revolution [158]. Our ancestors were able to consume and survive thanks to the gut microorganisms that broke down plant fibre. The acts that the enzymes of the gut microbiota can carry out on compounds that are not digested by human enzymes and end up in the GI tract include fermentation, hydrolysis, denitrification, sulphate reduction, and aromatic fission [159]. Our bodies can now digest simple and complex carbohydrates as well as a variety of other foods on their own, thanks to the presence of these nutrients. This seeming mismatch in evolution seems to increase the risk of cancer by increasing sugar consumption [160]. Several of the plant components associated with improved health can be converted by the gut microbiota into bioactive substances, including SCFAs and bioactive phytochemicals. SCFAs (acetate, propionate, and butyrate) that enter the gut microbiota are produced by the fermentation and hydrolysis of complex carbohydrates and dietary fibre [128]. For instance, propionate controls glucose and lipid metabolism in the liver, whereas butyrate is a crucial fuel source for enterocytes in the gut [161]. Butyrate can also cause cell differentiation, death, and hyperacetylation of histones. Even though butyrate’s advantages are meant to stop cancer from starting and spreading, they seem to be affected by the host genotype and SCFA concentrations [162]. A decreased risk of developing cancer has also been associated with plant compounds, such as polyphenols, flavonoids, and glucosinolates [163,164]. Some bacteria can convert the glucosinolates found in cruciferous vegetables into anti-cancer isothiocyanates. Microbes that digest starch and dietary fibres include *Eggerthella* spp., *Alistipes putredinis*, *Eubacterium hallii*, and *Phascolarctobacterium faecium*. Starch and dietary fibres are broken down by *Eggerthella* and *Alistipes* [165,166]. Although it has been demonstrated that eating foods poor in fibre and hence deficient in polyphenols increases microbial pathogenicity and decreases barrier function [167], the connection between this and an increased risk of cancer is yet unknown [159]. According to a recent meta-analysis, flavonoids including quercetine and apigenin, may lower the risk of developing cancer. The capacity of gut microbiota to access downstream metabolites and nutrients has been connected to toxic and protective pathways associated with isoflavones in soy [163,168,169].

Amino acids and proteins can be converted into organic acids, such as phenols, indoles, amines, sulphur compounds, ammonia, and amines [170]. A variety of processes, including fermentation, deamination, decarboxylation, hydrolysis, and elimination, produce these by-products. Fatty acids and other lipids can also be broken down by digestive microbes, especially for the production of bile acids [128,171]. These conversions have been associated with cancer and impact both hepatic signalling and gut microbiota [172,173]. It has been shown that high-protein, high-fat diets cause levels of carcinogenic fatty acids, such as N-nitroso compounds, to increase [174,175]. In addition, the production of secondary bile acids has increased, which has led to an increase in the variety of microorganisms that break down plant polysaccharides in animal diets [176]. When low-carbohydrate diets are paired with high-protein diets, butyrate production and beneficial *Roseburia/Eubacterium rectal* levels in the faeces are lowered [175]. Increased fibre intake did not significantly lower the incidence of colon cancer compared to a reduction in animal products [177]. These results suggest that a diet high in carbohydrates and low in protein may reduce the incidence of cancer.

The quality of our foods has drastically declined compared to hunter-gatherer societies. We eat food that is more processed, has fewer vitamins, fibre, and more pollutants and is more inflammatory [178,179]. Moreover, we consume more calories, and obesity rates are at an all-time high. Inflammation rises with increased body fat [180,181], and immune system suppression occurs with increased body fat [182,183]. Of other primate species, industrialized humans have the most minor variety and number of gastrointestinal bacteria [184]. According to Rogers [185] and Elinav et al. [186], changed gut microbiomes can lead to increased inflammation, altered immunosurveillance, and altered metabolism [187], which can subsequently result in genomic instability and accelerated cell division [188]. Although any of these elements may not cause cancer on their own, when taken as a whole, they induce cellular stress, which may cause the growth of cancer. In each of these situations, abandoning a hunter-gatherer lifestyle may increase cancer risk by altering hormone levels [149]. In particular, the existence of certain bacteria may influence the likelihood of developing cancer [189]. For instance, numerous viruses and harmful bacteria, such as the human papillomavirus, Epstein–Barr virus, *Helicobacter pylori*, and *Fusobacterium nucleatum,* are responsible for around 20% of the world’s cancer burden [155,190]. In addition, Cafiero et al. [191] observed that *Firmicutes* phylum abundances were significantly different in cancer stool samples compared to healthy or adenoma samples [192], with a higher presence of *Clostridium difficile*. These data assume particular interest in preventing *Clostridium difficile* infection, where faecal microbiome transplantation can occur in extreme conditions [193]. Additional microorganisms that could be employed as diagnostic bacterial markers include *A. listipes finegoldii*, *Bacteroides fragilis*, *Parvimonas micra*, *Porphyromonas asaccharolytica*, *Prevotella intermedia*, and *Thermanaerovibrio acidaminovorans* [189]. The observations described by Cafiero et al. [191] have two possible implications concerning gut dysbiosis. First, dysbiosis in the microbiome linked to blood faeces presence might also be used to explore the underlying reasons for different patterns of mortality in different populations across the world. Second, the proper prebiotics/probiotics intervention could modify microbiome dysbiosis and possibly blood faeces biomarkers to reduce the risk of premature mortality [191].

**Figure 5:**
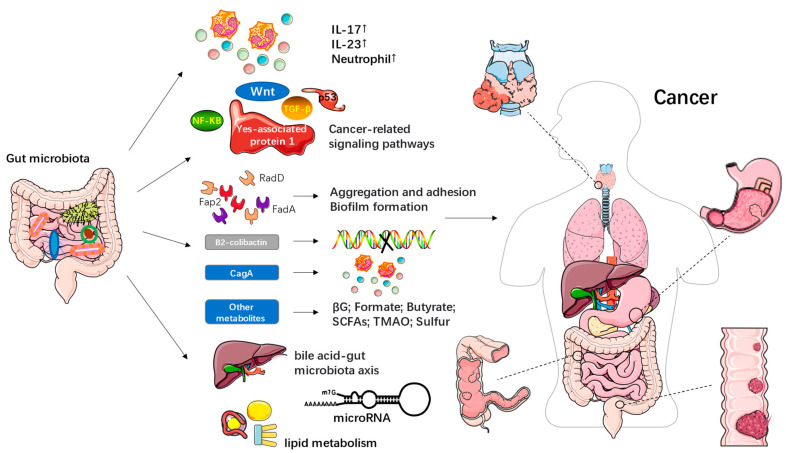
The role of the gut microbiota in the incidence and progression of cancer. Reprinted from Li et al. [195] under the terms and conditions of the Creative Commons Attribution (CC BY) license (https://creativecommons.org/licenses/by/4.0/).

The microbiome-immune crosstalk mechanisms during cancer initiation and progression are illustrated in Figure 5. This relationship is frequently described by two basic mechanisms: 1. Microbes that directly influence anti-tumour effectors by acting as antigens 2. Indirect effect via adjuvant cues provided by released by-products or induction of cytokine production (Figure 5). Mucosal microbes can modulate the immune system locally or after translocating to the sites of growing tumours. Moreover, they are able to transmit their influences to distant sites using mediators like metabolites, cytokines, chemokines, toxins and vesicles. Microbes can either interact directly with immune cells or provide indirect adjuvant cues for immunomodulation. The consequent inflammation can be either pro-tumorigenic or anti-tumorigenic, with a diverse range of effects on the innate and the adaptive immune system [194].

### 4.3. Food System and Gut Microbiota Transition in Cardiovascular Disease

According to Roth et al. [196], CVD is the leading cause of mortality globally. The most prevalent cause of CVD, atherosclerosis, is brought on by a complicated chain of processes inside the artery wall, including rheology, lipid metabolism, and inflammation [197]. End-organ ischemia, thromboembolic infarction, and necrosis are brought on by the stenoses that develop in the coronary, renal, precerebral, and peripheral arteries. According to Yancy et al. [198], heart failure (HF) is a condition brought on by the heart’s decreased capacity to fill or evacuate blood. HF may be brought on by any condition that compromises the anatomical or functional integrity of the heart, including valvular, coronary, or myocardial illness. Certain microbiota traits, such as a reduction in the number of microorganisms with the ability to produce butyrate and an increase in the levels of the microbiota- and diet-dependent metabolites trimethylamine-N-oxide (TMAO), have been consistently seen in both disorders [199].

**Figure 6:**
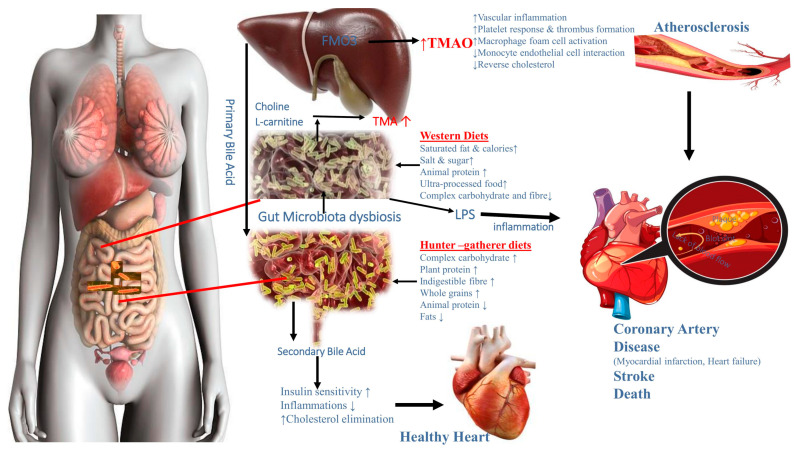
Food System-gut-heart proposed mechanisms of interactions. FMO3; flavin-containing monooxygenase 3, LPS; lipopolysaccharide, TMA; trimetylamine, TMAO; trimethylamine-N-oxide.

The exceptional cardiovascular health of communities that once hunted, fished and practised subsistence farming is noteworthy [90,115,200,201]. Even among individuals aged 60 years and older, heart and vascular disease fatalities in these populations are rare [115,202]. The contrast between rural and industrialised populations is particularly pronounced at older ages. Less than 30% of hunter-gatherers and subsistence farmers in the US who are 60 years or older have even moderate hypertension, compared to more than 60% of hypertensive US individuals [203,204]. According to Trøseid et al. [205], interactions between nutrition and the gut microbiota may have a combined or independent effect on atherosclerosis, acute coronary syndromes, and heart failure. Red meat-heavy Westernized cuisine encourages the creation of TMA by bacteria, which is then oxidised in the liver to produce the pro-atherogenic metabolite TMAO. By interfering with the transfer of cholesterol, the development of foam cells, and platelet aggregation—the latter of which may have an impact on acute coronary syndromes—TMAO may contribute to atherosclerosis [205]. The short-chain fatty acid butyrate, which has immune-modulatory effects on the gut mucosa and also serves as the primary energy source for colonocytes, is produced by bacteria and is influenced by dietary fibre levels. Reduced butyrate levels in the gut may exacerbate dysbiosis, exacerbate local inflammation, and lead to decreased gut barrier function, which may allow bacterial toxins such as lipopolysaccharide (LPS) to seep out and exacerbate both local and systemic inflammation [205] (Figure 6).

According to several studies, patients with coronary heart disease and those who are symptomatic have lower proportions of Bacteroidetes and lower abundances of *Roseburia Intestinalis* and *Faecalibacterium prausnitzii*, *Eubacteriumrectale*, known producers of the SCFA butyrate, and higher proportions of *Firmicutes*, several *Streptococcus* species and genera of the Enterobacteriaceae family, Escherichia-Shigella [206,207,208]. Additionally, numerous studies have noted decreased levels of *Faecalibacterium* from the *Ruminococcaceae* family [209], *Faecalibacterium prausnitzii* [210], *Eubacterium rectale* from the *Lachnospiracea* family, and *Blautia* from the *Lachnospiracea* family on the genus level [211], as well as decreased relative abundances of these species in patients with HF. The relative decrease in taxa from the *Lachnospiracea* or *Ruminococcacea* families, which are well known for their ability to produce butyrate, was a recurrent result in the research. The major source of energy for colonocytes to maintain the gut mucosal barrier is butyrate and other SCFAs, which are the by-products of the fermentation of dietary fibres [199,212]. In addition, butyrate has local anti-inflammatory effects in the intestinal mucosa via activating colonic regulatory T cells, suggesting that gut microbial alterations impacting butyrate may also affect inflammatory pathways [213]. Loss of butyrate-producing bacteria may cause the gut mucosal barrier to become dysfunctional, allowing microbial toxins such as LPS to leak out and cause inflammation by binding to Toll-like receptors and other innate immune system receptors [39,214].

### 4.4. The Underlying Mechanisms of the Link between the Food System and the Gut Microbiota Transition in Obesity, Cardiovascular Disease, and Cancer

The most significant modification in the host–microbiota symbiotic relationship occurred about 10,000 years ago, during the Neolithic revolution, sometimes known as the “agricultural revolution” [94,215]. This revolution is built on the transition from hunting and gathering to agriculture and permanent towns. Agriculture and animal husbandry evolved naturally throughout this time, resulting in natural changes in human lifestyle and the formation of present human genomes [216]. Because of its extraordinary plasticity, GM may change its composition and adapt to diet/food availability, and the evolution of agricultural societies may have promoted the creation of microbial communities capable of digesting complex substrates such as polysaccharides [111]. Indeed, agricultural communities derive the majority of their daily energy from a single cooked cereal grain [203]. In this sense, the selection forces to which Homo sapiens were subjected, particularly those linked with the dietary environment, including those caused by microbiota, may have been substantially changed. Numerous studies have been conducted on the metabolic genetic adaptations to this nutritional change brought on by the Neolithic transition [204,217,218,219]. However, in addition to any genetic changes brought about by altered intakes and ratios of macro- and micronutrients, the switch to agriculture may have also affected the proportions of microbial species consumed by Homo sapiens as well as the amount of non-digestible food in its diet, upsetting the ecological niches occupied by the microbiota. Changes in carbohydrate consumption appear to have shifted the balance between ecological niches of the oral microbiota in favour of cariogenic bacteria, as evidenced by the sequencing of calcified dental plaque from ancient teeth [220]. Additionally, the increased prevalence of infectious diseases associated with the Neolithic transition, such as higher population density, sedentariness, and contact with domesticated animals, placed novel selection pressures on the immune system [221,222]. This adaptation may have changed the immune system’s responsiveness, particularly the tolerogenic response, to the microbiota and, consequently, the parameters for maintaining symbiosis [22].

The protection provided by the gut microbiome against pathogenic bacteria and the fermentation of dietary, complex (and hence indigestible to the host) plant polysaccharides and host-produced glycans (such as mucin) are two important functions of the gut microbiota. There is strong proof that the microbiome influences the immune system as a whole [223]. A complex web of interactions that includes metabolic, immunological, and neuroendocrine crosstalk among them regulates and stabilises the symbiotic connection between the gut microbiota and the host. Moreover, bidirectional neuroendocrine signalling and immunological activations have shown reciprocal connections between the gut microbiota and the brain, sometimes known as the “gut-brain axis” [224]. This crosstalk may be caused by metabolites produced by microorganisms, which have a variety of functions, including functioning as signalling molecules to control host neuro-immune-inflammatory axes that could physiologically connect the gut with other organ systems [15]. In addition to allowing for the tolerance of commensal bacteria and oral food antigens, the interaction between the microbiota and immune system at the gut level also helps the immune system identify and combat opportunistic bacteria, avoiding bacterial invasion and infection. These microbiotas have broader impacts and affect localized immune responses, supporting innate and adaptive immunity at many levels [225]. Because of this, gut microbiota dysbiosis is thought to increase obesity, endotoxemia (increased LPS production), intestinal permeability, energy generation (energy harvest), insulin resistance, and pro-inflammatory cytokine production [28].

The gut microbiota and its associated metabolites can interfere with the host’s regular cell cycle, causing changes in the cell and protein expression that regulate cell division, DNA repair, and apoptosis, according to research by Gharaibeh et al. [226] and Fellows et al. [227]. Additionally, it has been demonstrated that the gut microbiota can affect host systemic inflammation and immune homeostasis, increasing the susceptibility to malignant tumours and affecting the clinical immunotherapy response of tumours [228]. In addition to maintaining the homeostasis of several T cell populations in the gut, including regulatory T cells (Treg), T helper 1 (Th1) and T helper 17 (Th17) cells [229,230], as well as mucosal-associated invariant T cells [231], there is evidence that the gut microbiota plays a significant role in triggering the production of immunoglobulin A [232,233]. The intestinal microbiota produces bioactive small molecule metabolites [234]. Examples include amino acid metabolites [235,236], lipids (such as N-acyl amides) [237], derivatives of carbohydrates, including SCFAs, and modifications to bile acids [238]. Many of these metabolites have an impact on mammalian physiology as ligands for nuclear hormone receptors and G-protein coupled receptors [239,240,241], which could be targeted for small molecule drugs [242] to treat and/or prevent diseases like autism [243,244], diabetes [245], inflammatory bowel disease [213], and coronary vascular disease [246,247].

**Table 1:** Impact of food systems and gut microbiota transition on host and NCD (↑: increased; ↓: decreased).

Food Systems	Dietary Pattern	Impact on Gut Microbiota Diversity	Impact on Host	Reference
Food System 1 Hunter-gatherer (Palaeolithic diet)	Plant (fruits, roots, legumes, nuts, and other non-cereals)	↑* Clostridium * ↑* Bacteroides* ↑* Verrucomicrobia * ↑* Mollicutes ↑ Aeromonadaceae* ↑* Oxalobacteraceae* ↑* Methanomassiliicoccaceae* ↑* Prevotella ↑ Catenibacterium * ↑* Eubacterium* ↑* Lachnospira* ↑* Treponema * ↑* Succinivibrio * ↑* Treponema * ↑* Eubacterium* ↑* Blautia* ↑* Dorea * ↑* Eubacterium* ↓ *Firmicutes* ↓ *E. coli*	↓ Visceral fat ↓ Body mass ↓ Inflammation ↑ Promote gut barrier integrity via anti-tumorigenesis ↑ SCFA synthesis ↑ Insulin sensitivity ↓ Obesity ↓ Cancer ↓ Cardiovascular diseases	[24,56,107,248,249,250,251,252,253,254]
Food System 2 (Neolithic revolution)	Agricultural diets, predominantly containing plant-based components with the presence of animal-based components	↑ Prevotella ↓ Bacteroides ↑ C. clostridioforme ↑ Faecalibacterium prausnitzii ↑ Firmicutes ↑* Capnocytophaga endotelialis* ↑* Capnocytophaga haemolytica* ↑* Capnocytophaga ochracea* ↑* Capnocytophaga sputigena* ↑* Eikenella corrodens*	Body mass Body fat ↑ Visceral fat ↑ Insulin sensitivity	[254,255]
Food system 3	Grains and carbohydrates. low in carbohydrates and rich in animal fats and proteins	↓* Prevotella* ↓* Akkermansia* ↓* Muciniphila* ↑* Proteobacteria* ↑* Firmicutes* ↓* Bacteroidetes* ↑* Anaerotruncus genus* ↑* Eisenbergiella * ↑* Lachnospiraceae* ↑* Campylobacter* ↑* Flavonifractor* ↑* Erysipelatoclostridium* ↑* aecalibacterium* ↑* Sutterella* ↑* Clostridium* ↓* Bifidobacterium* ↓* Roseburia*	Correlations with obesity Weight gain Cancer CVD ↓ Gut microbiome diversity ↓ SCFA synthesis ↑ Formation of nitrogen compounds	[225,248,249,250,254,256,257,258,259,260]
Food System 4 and 5	Western Diet	↑* Firmicutes ↑ Enterobacteriaceae * ↓* Actinobacteria * ↓* Prevotella* ↓* Akkermansia muciniphila* ↓* Faecalibacterium prausnitzii* ↓* Roseburia* spp., ↓* Eubacterium hallii * ↓* Clostridium clusters XIVa* and *IV* ↓* Ruminococcus* ↑* E. coli* ↑* Alistipes* ↑* Bilophila* ↑* Bacteroides* ↓* Roseburia* ↓* Eubacterium rectale* ↓* Ruminococcus bromii* ↑* Acinetobacter* ↑* Blautia* ↑* Dorea* ↑* Lactococcus* ↑* Proteobacteria* ↓* Bacteroidetes*	↑ Diabetes ↑ Allergies ↑ Cardiovascular disease and neurological disorders ↑ Dysbiosis ↑ Inflammation ↑ Obesity ↑ Inflammatory bowel disease Bacterial overgrowth associated with obesity ↑ Production of endogenous ethanol ↑ The risk of non-alcoholic fatty liver disease ↑ Pro-inflammatory properties promoting metabolic endotoxemia and low-grade inflammation	[176,254,261,262,263,264,265]
Food System 6	Vegan diet Probiotics Prebiotic Fermented foods	↑ All gut diversity	↓ Inflammation Body mass maintenance	[254,266,267]

## 5. Transformative Solution: Healthy Gut Microbiota Reversal Via Healthy Diets from Sustainable Food Systems Transformation

A “triple disaster” has been labelled the current state of agriculture and food systems in which obesity, undernutrition, and climate change are destroying both human and planetary health [268]. The main driver of the rising interest in changing the food system is the realization that the interconnected issues of poverty, malnutrition, environmental degradation, and climate change cannot be resolved through isolated interventions, but instead require a fundamental shift in the dynamics of food systems [269,270]. Comprehensive solutions must be developed to improve food availability, access, safety, affordability, and appeal to address the triple issue of malnutrition, hunger, micronutrient deficiencies, and obesity. An enabling environment of institutions, policies, rules, regulations, and investments that are coordinated and complementary across sectors is required to support this transformation of food systems [271].

By 2050, significant dietary changes will be necessary to transition to healthy diets. Maintaining a healthy gut microbiota is essential and achieved through eating smarter, one of the four pillars of transforming food systems [14]. Global demand for plant-based goods is rising as more people become aware of how much our dietary choices have an impact on our health. The substantial connections between excessive meat intake and the development of non-communicable illnesses, including obesity, type 2 diabetes, CVD, and some types of cancer, are still being revealed by researchers [272,273,274]. On the other hand, there is growing evidence that diets with higher plant-based food consumption are healthier and lower the risk of developing several chronic diseases [275], and that the high fibre and polyphenol content of plant-based diets encourages changes in gut microbiota composition that are thought to be responsible for these favourable health outcomes [276,277,278,279]. In addition, lessening one’s meat intake is increasingly regarded as a healthier and more moral choice. As a result, more and more flexitarian consumers are seeking plant-based meat alternatives to replace at least some of the animal meat they already eat [280].

As a result, the intake of items such as red meat and sugar would need to drop by more than 50%, while the consumption of fruits, vegetables, nuts, and legumes would need to treble. A diet high in plant-based foods and low in meals derived from animals helps the environment and human health [281]. An ideal calorie intake is part of a healthy diet, which also consists mostly of a variety of plant-based foods, little food derived from animals, unsaturated fats rather than saturated fats, small amounts of refined grains, highly processed foods, and added sugars. Diets that support long-term health and high nutritional status for a person as well as the community, and at the same time have a low environmental impact, are referred to as sustainable diets [282]. This definition encompasses both a viewpoint on environmental sustainability that considers all aspects of the environment, food production, economics, and society, as well as good nutrition that focuses on individuals, dietary consumption, and health consequences [283,284]. Although sustainable diets, often referred to as the planetary health diet, are based on health concerns and are consistent with many historic eating habits, they do not suggest that everyone in the world should consume the same foods or follow a specific diet. The Planetary Health Diet, on the other hand, offers empirical food groupings and intake ranges that, when integrated with a diet, would improve human health [281]. The globally applicable planetary health diet must be interpreted and adapted locally, and it should do so in a way that takes into account local culture, geography, and demographics. In line with the concept of “food and nutrition security,” sustainable diets should be available to most people, ideally be based on locally grown foods and ingredients, and be priced reasonably [285,286]. According to Magkos et al. [286], the SHARP diet concept has been put forth to operationalize sustainable diets. The acronym stands for Sustainable (S), Healthy (H), Affordable (A; accessible for consumers while also supporting the agriculture food sector), Reliable (R; stable in its supply and safe), and Preferable (P; consistent with cultural norms and food preferences) [274].

### Shreds of Evidence of Transformative Dietary Pattern Solutions

While it has already been shown that geographical location, culture, and genetic background all influence microbiota composition [55], some scientists believe that nutrition accounts for more than 50% of microbial diversity [287,288]. Despite the difficulty of precisely determining this value, there is evidence that dietary treatments with considerable changes in content can impose modulatory impacts on microbiota composition that can be detected within 1–4 days and are robust enough to modify the enterotype [289,290]. Nonetheless, when the diet is stopped, the dietary modulatory effects fade over time, and the body returns to its former condition [287]. All of these research studies support the notion that interventions to alter gut microbiota must be ongoing. In accordance with this, using diet as a modulatory technique may be beneficial when diet is viewed as a long-term modification of daily routines [55]. Hence, the most important factors in preserving and sustaining a healthy life are a balanced diet and a variety of foods. There is no shortage of evidence for a balanced eating lifestyle that ensures the persistence of healthy gut microbiota in the world’s varied food systems (Table 1). The Dietary Approaches to Stop Hypertension (DASH), the Mediterranean diet (MD) and Nordic diets (ND) are well-known dietary patterns that have been conclusively linked to longevity and good health (or lower mortality and risk of NCDs) in the general population [291,292]. The DASH diet focuses on low-fat and nonfat dairy products and consists primarily of plant-based meals with some animal components. The MD and ND emphasize locally produced foods, since they both incorporate traditional dietary patterns [286]. The MD was created to capture the usual eating patterns of people living in the Mediterranean region in the early 1960s, which included a high intake of plant-based foods, a low intake of meals derived from animals, and a moderate intake of alcohol [286,293]. The MD model promotes the composition of the gut microbiota for human health, as noted in previous research [28]. One of the dietary approaches that is based on plants is the MD. The MD is an eating style that offers a wide variety of foods, satisfies dietary needs, and maintains consumer health [294,295]. The traditional MD plan calls for a high intake of fruits, vegetables, beans, nuts, seeds, whole grains, fish, other seafood, olive oil, dairy products (primarily yogurt and cheese), and whole grains [296]. On the other hand, according to Evert et al. [297], it contains small amounts of red meat, sweets, or honey, and small to moderate amounts of alcohol. The ND, which highly emphasizes health and ethical production philosophy, was created in 2004 as a novel way to approach traditional foods [298]. The high consumption of plant-based foods, whole grains, nuts, dairy, fish, shellfish, free-range meat, and the game is another characteristic of this diet [286,299]. Generally, the MD and ND are viewed as models of a balanced regional omnivorous diet that takes into account environmental factors, food culture, health, and taste [286]. The Mediterranean diet is linked to increased SCFA synthesis in the stomach [38,300]. These compounds serve critical functions in preserving the integrity of the large intestine and small intestinal barrier, giving energy to epithelial cells, and lowering inflammation [38,55], as well as promoting greater microbial diversity in the gut [300,301].

Other transformative dietary pattern solutions that are gaining traction include the use of prebiotics and probiotics. The use of prebiotics is possibly the most well-researched technique for altering the microbiota [55]. Prebiotics are described as “substrate that is selectively utilised by host microorganisms delivering a health benefit” [302], which are nutrients that are resistant to stomach acid secretion and digestive enzymes that, once in the gut, increase the development or activity of beneficial bacteria [55]. Certain dietary components have been studied as prebiotics, including inulin, fructooligosaccharides (FOS), galactooligosaccharides (GOS), and resistant starch (RS), and their efficacy is commonly measured indirectly by the production of short-chain fatty acids (SCFAs) or a decrease in intestinal pH [230,247]. Inulin promotes the proliferation of lactobacilli and bifidobacteria; moreover, a rise in F. prausnitzii and A. muciniphila populations in the gut has been reported, which appears to generate early satiety by altering gut endocrine activity [55]. Nonetheless, determining the mechanisms behind these impacts remains difficult [303]. FOS are oligosaccharides of glucose and fructose that vary from inulin, in that they have a polymerization degree of 2 to 8. GOS are oligosaccharides of glucose and galactose that have a polymerization degree of 2 to 8. FOS and GOS, like typical prebiotics, have been used to promote the development of beneficial bacteria, such as bifidobacteria and lactobacilli. FOS supplementation resulted in an increase in Bifidobacterium and F. prausnitzii in culture-dependent research, but high-throughput sequencing indicated alterations in over 100 bacterial taxa in a culture-independent investigation [55]. The most significant changes in abundance were an increase in Bifidobacterium, decreases in the genera *Phascolarctobacterium*, *Enterobacter*, *Turicibacter*, *Coprococcus*, and *Salmonella*, a rise in Bacteroidetes overall and a drop in the phylum Firmicutes [304]. RS is defined as the total quantity of starch and starch breakdown products that resist digestion and has been demonstrated to be made up of a linear molecule of −1, 4-D-glucan produced from the retrograded amylose fraction [55]. Notably, an increase in RS in the diet has been linked to greater levels of colonisation by the phylum Bacteroidetes and the genera Bifidobacterium, *Akkermansia*, and *Allobactum* [305].

The area of probiotics—living bacteria that, when supplied in sufficient proportions, offer a health benefit to the host [176,306]—has developed significantly in recent years. The yeast Saccharomyces cerevisiae and members of the bacterial genera Lactobacillus and Bifidobacterium are often used as probiotics, while certain formulations may also include *Streptococcus*, *Enterococcus*, *Pediococcus*, *Propionibacterium*, *Bacillus*, or *Escherichia* strains. Most Lactobacillus and Bifidobacterium species have been designated as “Generally Recognised as Safe” by the US Food and Drug Administration and “Qualified Presumption of Safety” by the European Food Safety Authority, allowing them to be used as probiotics preferentially [55]. On the other hand, their long history of usage as probiotics means that there is a considerable body of evidence indicating a wide range of beneficial qualities [307]. However, it is important to note that probiotic effects are strain-specific, not species-specific [308]. Nonetheless, it is envisaged that other species will be employed as probiotics in the near future—species that are more often found in the human gut and play essential roles in alleviating intestinal inflammation, triggering immunological modulation, or boosting intestinal barrier function [55]. These are expected to include anti-inflammatory bacteria (*A. muciniphila* and *F. prausnitzii*) as well as butyrate-producing bacteria [306,309].

## 6. Conclusions and Perspectives

During the past few decades, it has been clear how crucial the gut microbiota is in the relationship between nutrition and human health. After reviewing recently discussed aspects of the transformation of the food system, it can be said that obesity, metabolic syndrome, and NCDs are somewhat connected to the shifts in the food system brought about by civilization, urbanization, population expansion, and technological improvement. There is evidence that the gut microbiota is affected or disturbed in some way by all stages of the food system transformation. It has been demonstrated that highly processed diets that are low in micronutrients, dietary fibres, and other beneficial components limit bacterial diversity. Disrupted microbial functioning has also been described. These results suggest that the association between processed food items and metabolic syndrome may involve disturbance of the gut microbiota as a potential mechanism. Yet, it has been demonstrated that consuming plant-based foods and leading a sustainable dietary lifestyle, which is characteristic of our ancestors’ hunter-gatherer dietary patterns, help to preserve and improve gut health.

Dysbiosis is frequently defined by decreased microbial diversity, declining percentages of *Bifidobacteria* and *Lactobacilli*, and increased pathogen survival, but the balance of the gut ecosystem is probably a much more nuanced idea, and more thorough profiles of disturbed or healthy gut microbiota still need to be established. Overall, it is possible to say that there is evidence from a variety of research avenues that changes in the food system through the advent of agricultural transformation and food processing technology change the nutritional content of food items in a way that might account for their connection to the present epidemic of obesity, cancer, and cardiovascular diseases. These effects appear to be mediated in large part by the gut microbiome transition. These findings, along with increased public awareness of this issue, might provide the food sector and regulatory bodies with fresh motivation to create sustainable diets and science-based public health policies. The food business may begin emphasizing the provision of more foods with low-calorie densities and the best possible preservation of micronutrients. Hence, certain harmful industrial methods may be changed, removed, or replaced with less harmful ones. Translation of scientific results into intelligible nutritional advice and adequate, clear, and thorough public communication is key to changing the existing scenario. When all these are done, consumers will better be positioned to make informed dietary choices so that it may be possible to limit the rate of increase in obesity, cancer, and cardiovascular disease prevalence.

However, thorough further human-based research is required, investigations of the intricate interactions that occur between food system and microbiota are required to successfully influence desirable changes in human microbiota or modify its aberrant composition in disease.

## Data Availability

The datasets generated for this study are available on request to the corresponding author.
